# Successful Management of Heterotopic Cervical Pregnancy After In-Vitro Fertilization Presenting With Hyperemesis Gravidarum: A Case Report

**DOI:** 10.7759/cureus.54612

**Published:** 2024-02-21

**Authors:** Chalent Alexakis, Konstantinos Zacharis, Asimina Paraskevi Barmparousi, Stavros Kravvaritis, Theodoros Charitos

**Affiliations:** 1 Department of Obstetrics and Gynaecology, General Hospital of Lamia, Lamia, GRC

**Keywords:** ectopic pregnancy, heterotopic cervical pregnancy, conservative management, hyperemesis gravidarum, in-vitro fertilization

## Abstract

Heterotopic pregnancy, defined by the simultaneous existence of intrauterine and ectopic pregnancy, is a rare pathological condition. In women undergoing assisted reproduction techniques, the frequency is higher. The possibility of the simultaneous existence of an ectopic pregnancy, when the certainty of an endometrial pregnancy has been assured, should always be considered with special care. The case of a patient with heterotopic pregnancy with assisted conception and the only symptom being nausea and vomiting is presented.

## Introduction

Heterotopic cervical pregnancy (HCP) is an uncommon type of ectopic pregnancy. It is a circumstance where at least one gestational sac in the uterus co-exists with at least one sac in the cervical canal [1]. The first literary description of this phenomenon was in 1708 as an autopsy finding [2]. The incidence of HCP (for spontaneous pregnancy) is reported to be one in every 30,000 pregnancies. However, due to the use of artificial reproductive technology, this ratio is estimated to be one in 100 in-vitro fertilization (IVF) recipients [3]. Several approaches have been described for the management of this type of pregnancy, with the main goal being fertility preservation. Conservative treatment includes methotrexate or transvaginal potassium chloride injection, while surgical methods are uterine artery ligation and embolization, cervical curettage with/without cerclage, and Foley catheter insertion [4]. We hereby present a case of an early HCP presenting with hyperemesis gravidarum that was managed successfully after KCl (potassium chloride) injection for pregnancy reduction.

## Case presentation

**Figure 1:**
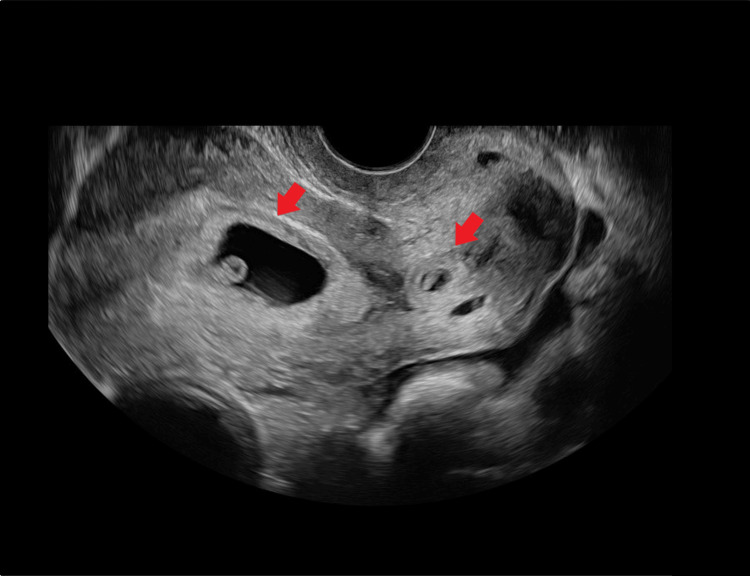
Transvaginal ultrasonography showed an intrauterine and a cervical pregnancy simultaneously at six weeks and four days of gestation Arrows are showing the two gestational sacs.

**Figure 2:**
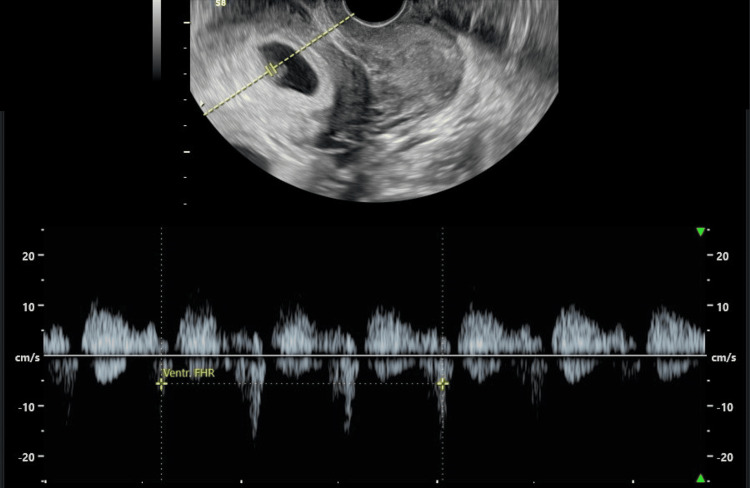
Gestational sac in the uterine cavity with viable fetus (FHR: 125 bpm)

**Figure 3:**
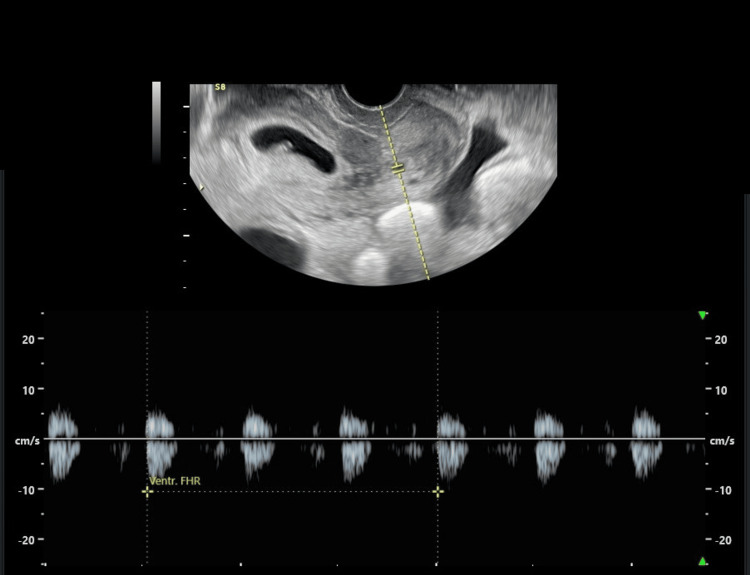
Gestational sac in the uterine cervix with viable fetus (FHR: 121 bpm)

A 33-year-old gravida 2 para 0 patient with a history of pregnancy after IVF-ET and transfer of two blastocysts was admitted to the obstetrical department for excessive nausea and vomiting, where the diagnosis of hyperemesis gravidarum was placed (3 plus ketones in urine). The patient was undergoing her sixth week of pregnancy. The first trimester transvaginal ultrasound showed a normoimplanted gestational sac with a live fetus of gestational age six weeks, with a fetal heart rate of 125 beats per minute (bpm). Subsequently, a second ectopic gestational sac was spotted endocervically with a viable fetus inside with a heart rate of 121 bpm (Figures 1-3). Hence, the diagnosis of HCP was established, and she was referred to a tertiary hospital for further management.

The patient was informed of her medical options, though she expressed her desire to maintain the intrauterine pregnancy if it remained viable. The available options were methotrexate injection, dilation and curettage, and KCl injection. After thorough counseling, the option of reduction of cervical pregnancy through KCl injection was chosen.

**Figure 4:**
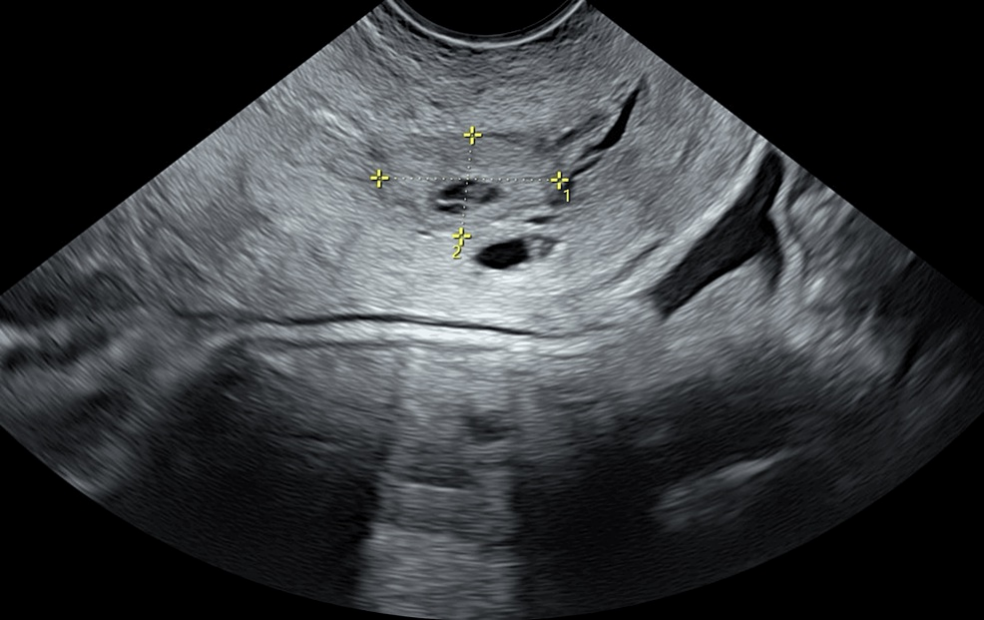
Transvaginal ultrasonography after KCl injection revealed that a hypervascular echogenic change was shown in the cervical pregnancy

At 6 weeks and 6 days of gestation, the patient underwent a selective reduction of the ectopic gestational sac with ultrasound-guided potassium chloride injection (2ml-2meq/ml)into the cervical sac. The procedure resulted in a successful reduction of the cervical pregnancy while preserving the intrauterine pregnancy, followed by discharge in stable condition after an overnight hospital stay (Figure 4). The symptoms regarding hyperemesis gravidarum gradually improved within the next few days, and two weeks later, at eight weeks and four days of gestation, transvaginal ultrasonography revealed the successful management of the heterotopic pregnancy, whereas the intrauterine pregnancy continued.

The patient’s prenatal follow-up proceeded in our outpatient department. She attended all her perinatal appointments, and the cervical length was reassuring in both the first- and second-trimester ultrasounds. At 36 weeks and six days of gestation, the pregnant woman presented in an active stage of labor where she delivered, through a vacuum-assisted vaginal delivery due to maternal exhaustion, a healthy female baby.

## Discussion

In ectopic cervical pregnancy, the implantation of the fertilized egg involves the epithelium of the endocervical cavity. Cervical pregnancy is not common. This is an extremely rare form of ectopic pregnancy that accounts for less than 1% of all ectopic pregnancies [3]. The clinical diagnosis of ectopic cervical pregnancy is not easy. Before the 1980s, the diagnosis of cervical pregnancy was usually histological, after failure to treat uncontrolled bleeding as trophoblastic vessels can reach and erode the thin wall of the cervix, resulting in the need to perform a hysterectomy [5]. The remarkable improvements that have been achieved in recent years in ultrasound imaging techniques, allowing the early diagnosis of cervical pregnancy, also provide the possibility for a more conservative treatment of the disease.

Although HCP is an event that occurs rarely, due to the more frequent usage of artificial reproductive techniques (ART), the incidence of HCP has increased [6]. This association is not well understood but could be explained by a correlation of risk factors: (1) an interconnection between risk factors which is common to patients undergoing this procedure (i.e., cervical irregularities and curettage procedures) and (2) reasons that are related to the method (cervical trauma during the process, volume, and viscosity of the transfer medium and reflux of the transferred embryo) [3]. Also, despite the numerous breakthroughs in the field of ART, the usage of ultrasound-guided embryo transfer increased the number of successful pregnancies; however, there was no effect noted on the incidence of heterotopic/ectopic pregnancy rates [7].

In medical literature, several alternative treatment approaches can be chosen based on patient presentation and preference. In a study by Moragianni et al., 39 cases were reported in the literature, of which 30 were a result of IVF, while the rest were products of natural conception [8]. The majority of cases are managed surgically by aspiration, extraction, hysteroscopy, and dilation and curettage (n = 16; 41%). Meanwhile, surgical intervention involves aspiration, extraction, hysteroscopy, and dilation and curettage (n = 14; 36%). The combination of these two methods was performed in a smaller percentage (n = 7; 18%). From the literature, three cases (8%) reported using a Foley catheter, while six (13%) used a cervical cerclage. Only in two cases (5%), the combination of the latter was used. In seven cases (18%), embolization of the uterine arteries was performed, while the pregnancy spontaneously resolved in one out of 39 cases (3%) [8].

## Conclusions

This case is a particular event that followed a unique course. Following a course of IVF, the patient developed an HCP, diagnosed via transvaginal ultrasound during a hospital admission due to hyperemesis gravidarum. The ectopic pregnancy was reduced at six weeks of gestation, while the intrauterine pregnancy remained viable, resulting in the successful delivery of a healthy child. The patient had no vaginal bleeding either before or after the reduction of the heterotopic pregnancy and was completely asymptomatic from complaints regarding cervical pregnancy. This case is an example of a conservatively managed HCP that can result in a live birth.

Prompt management of such a pregnancy is very important, especially for patients with known infertility, such as in this case report. Despite multiple treatment possibilities, there are currently no guidelines or recommended first-line techniques for the appropriate way to manage HCP due to minimal publications for such cases in international literature. Therefore, the treatment must be individualized and take into account the patient’s desire, whether they wish to attempt to maintain the intrauterine pregnancy, as well as the equipment and experience of the medical team.

## References

[1] Maleki A, Khalid N, Rajesh Patel C, El-Mahdi E (2021). The rising incidence of heterotopic pregnancy: current perspectives and associations with in-vitro fertilization. Eur J Obstet Gynecol Reprod Biol.

[2] Bright DA, Gaupp FB (1990). Heterotopic pregnancy: a reevaluation. J Am Board Fam Pract.

[3] Terra ME, Giordano LA, Giordano MV, Sá RA, Campos F, Yadid IM, Pinto FO (2019). Heterotopic cervical pregnancy after in-vitro fertilization - case report and literature review. JBRA Assist Reprod.

[4] Kim JW, Park HM, Lee WS, Yoon TK (2012). What is the best treatment of heterotopic cervical pregnancies for a successful pregnancy outcome?. Clin Exp Reprod Med.

[5] Alanis-Fuentes J, Brindis-Rodríguez A, Martínez-Arellano M (2015). Embarazo ectópico cervical. Tratamiento histeroscópico, presentación de un caso [Cervical ectopic pregnancy. Hysteroscopy treatment, case report]. Ginecol Obstet Mex.

[6] Gyamfi C, Cohen S, Stone JL (2004). Maternal complication of cervical heterotopic pregnancy after successful potassium chloride fetal reduction. Fertil Steril.

[7] Sallam HN, Sadek SS (2003). Ultrasound-guided embryo transfer: a meta-analysis of randomized controlled trials. Fertil Steril.

[8] Moragianni VA, Hamar BD, McArdle C, Ryley DA (2012). Management of a cervical heterotopic pregnancy presenting with first-trimester bleeding: case report and review of the literature. Fertil Steril.

